# Outcomes of COVID-19-Associated Hospitalizations in Geriatric Patients with Dementia in the United States: A Propensity Score Matched Analysis

**DOI:** 10.3390/geriatrics9010007

**Published:** 2024-01-05

**Authors:** Tomas Escobar Gil, Mohammed A. Quazi, Tushita Verma, Amir H. Sohail, Hafiz Abdullah Ikram, Adeel Nasrullah, Karthik Gangu, Asif Farooq, Abu Baker Sheikh

**Affiliations:** 1 Department of Internal Medicine, University of New Mexico, Albuquerque, NM 87106, USA; tescobargil@salud.unm.edu (T.E.G.); tverma@salud.unm.edu (T.V.); haikram@unm.edu (H.A.I.); 2 Department of Mathematics and Statistics, University of New Mexico, Albuquerque, NM 87106, USA; 3 Division of Surgical Oncology, University of New Mexico, Albuquerque, NM 87106, USA; asohail@salud.unm.edu; 4 Division of Pulmonology and Critical Care, Allegheny Health Network, Pittsburg, PA 15212, USA; adeel.nasrullah@ahn.org; 5 Department of Internal Medicine, University of Kansas Medical Center, Kansas City, KS 66160, USA; kgangu2@kumc.edu; 6 Department of Family and Community Medicine, Texas Tech Health Sciences Center, Lubbock, TX 79409, USA; afarooq76@gmail.com

**Keywords:** COVID-19, dementia, geriatrics, mortality, prevalence, complications, United States, national inpatient sample

## Abstract

Previous studies have convincingly demonstrated the negative impact of dementia on overall health outcomes. In the context of the COVID-19 pandemic, there is burgeoning evidence suggesting a possible association between dementia and adverse outcomes, however the relationship has not been conclusively established. We conducted a retrospective cohort study involving 816,960 hospitalized COVID-19 patients aged 65 or older from the 2020 national inpatient sample. The cohort was bifurcated into patients with dementia (*n* = 180,845) and those without (*n* = 636,115). Multivariate regression and propensity score matched analyses (PSM) assessed in-hospital mortality and complications. We observed that COVID-19 patients with dementia had a notably higher risk of in-hospital mortality (23.1% vs. 18.6%; aOR = 1.2 [95% CI 1.1–1.2]). This elevated risk persisted even after PSM. Interestingly, dementia patients had a reduced risk of several acute in-hospital complications, including liver failure and sudden cardiac arrest. Nevertheless, they had longer hospital stays and lower total hospital charges. Our findings conclusively demonstrate that dementia patients face a heightened risk of mortality when hospitalized with COVID-19 but are less likely to experience certain complications. This complexity underscores the urgent need for individualized care strategies for this vulnerable group.

## 1. Introduction

The COVID-19 pandemic has, since its onset, highlighted the vulnerabilities of specific sub-populations, amplifying disparities in health outcomes [1]. Chief among these vulnerable groups are geriatric patients, with those diagnosed with dementia being especially at risk [2]. Dementia, a chronic neurodegenerative condition, is characterized by a progressive deterioration of cognitive functions and memory capabilities [3]. Globally, over 55 million people are affected by dementia, with an overwhelming 60% living in low- and middle-income countries [4]. The implications of dementia are not only cognitive but also economic; in 2019, the global cost attributed to dementia was approximately 1.3 trillion US dollars [4]. Furthermore, dementia is recognized as the seventh leading cause of death and a significant source of disability and dependency among older individuals [4].

Research indicates that elderly COVID-19 patients with dementia are especially vulnerable due to a combination of biological, psychological, and societal factors [5]. Commonly, these individuals have multiple coexisting health conditions, experience physical frailty, and have compromised immune responses, which together can lead to severe COVID-19 outcomes [5]. Their cognitive impairments can also impede their ability to understand, interpret, and adhere to preventive measures and treatment protocols, thereby increasing their risk of infection and adverse health outcomes [6].

The challenges dementia patients face during the pandemic are multifaceted. Factors such as age, comorbidities, cognitive decline, and communication challenges amplify their susceptibility to COVID-19. Moreover, the pandemic can worsen cognitive symptoms, increase delirium risks, and intensify feelings of social isolation, further undermining their mental and physical health [3,4,7].

Throughout the pandemic, the complex nature of dementia care has been accentuated. Protocols like enforced social distancing, shifts in healthcare delivery, and strict visitation policies in care settings have unintentionally intensified isolation for dementia patients, leading to broader negative health implications [3]. Emerging studies suggest a potential bidirectional relationship between COVID-19 and dementia, wherein the exacerbation of one could intensify the other, resulting in prolonged hospitalizations and elevated mortality rates [8].

The intersecting challenges of COVID-19 and dementia present a global dilemma that requires careful consideration by healthcare professionals, caregivers, and policymakers [7]. Deepening our understanding of how COVID-19 affects dementia patients is crucial, and such insights are pivotal when shaping clinical strategies and developing effective, patient-centered care paradigms [9]. Driven by this need, our study delves into the clinical outcomes of COVID-19 patients with a dementia diagnosis, contrasting these with results observed in outpatient dementia settings.

## 2. Materials and Methods

A retrospective examination was carried out using the 2020 national inpatient sample (NIS), which contains data on 816,960 COVID-19 patients aged 65 and over. Out of these, 180,845 (22.1%) were diagnosed with dementia. Utilizing multivariate regression and propensity score matched methods, in-hospital outcomes such as mortality, comorbidity, hospital costs, duration of hospitalization, and discharge details were compared between patients with and without dementia.

This NIS database contains anonymized billing and diagnostic details from collaborating hospitals in the United States [10]. As there are no direct “human subjects”, it is not subject to institutional review board oversight, aligning with federal guidelines [11]. Hospitalized COVID-19 patients aged 65 and up were included in the study. ICD-10 CM and procedure codes were essential to ascertain patient samples and associated conditions, with a detailed code list available in Supplementary Table S1.

The dataset captured diverse care facets, including outcomes during hospital stay and discharge data. Information was categorized as follows: patient details (e.g., age, race, insurance status), hospital characteristics (e.g., bed size, region), and severity of illness (e.g., length of stay and in-hospital complications). The study’s prime focus was in-hospital mortality, while secondary outcomes spanned various clinical inpatient complications and systemic indicators like mechanical ventilation use, length of stay, and hospitalization costs.

Analysis was performed using Python, with data curation facilitated by SAS. From the original sample of 6.47 million observations, post-weighting adjustments, the dataset expanded to roughly 32.3 million discharges for 2020. From these, 1.67 million were COVID-19 admissions. Due to data omissions, the study worked with 816,960 records. When subdividing based on dementia status, 636,115 were dementia-free, and 180,145 were dementia-diagnosed. Statistical tests, such as the chi-square test, were used to discern relationships between cohorts. Linear regression focused on continuous outcomes like hospital stay length, while logistic regression catered to binary outcomes, including specific clinical complications.

## 3. Results

### 3.1. Demographics and Baseline Comorbidities

Of the 816,960 hospitalized COVID-19 patients we studied, 180,845 (22.1%) were also diagnosed with dementia. Females constituted a majority within the dementia cohort at 55.6%, and the highest dementia prevalence was found in patients aged 80 and above at 65.9%. Average ages stood at 82.8 years for females and 81.1 for males. This group was less represented in the median household income bracket below USD 50,000 at 30.5%.

Compared with the non-dementia cohort, those with COVID-19 and dementia exhibited lower percentages in smoking, alcohol consumption, drug use history, and several chronic conditions, including pulmonary disease, myocardial infarction, diabetes mellitus, cancer, and autoimmune diseases. On the other hand, they were more prone to hypertension, peripheral artery disease, chronic kidney disease, hypothyroidism, and depression. Both cohorts showed comparable rates for coronary artery disease. A significant portion of this patient group was treated at urban teaching hospitals (70.2%) and were predominantly Medicare beneficiaries (89.6%). For a comprehensive breakdown of patient demographics, see Table 1. Additionally, Supplementary Table S2 provides characteristics of the matched cohort post-propensity scoring.

### 3.2. In-Hospital Mortality

When considering factors such as age, gender, race, income, insurance status and more, our multivariate logistic regression analysis revealed a notable rise in in-hospital mortality for COVID-19 and dementia in comparison with COVID-19 and non-dementia patients (23.1% vs. 18.6%; *p* < 0.001). This difference was statistically significant, with an adjusted odds ratio (aOR) of 1.2 (95% CI 1.1–1.2) (Table 2). Our propensity score matching further supported this observation with an aOR of 1.2 (95% CI 1.1–1.2, *p*-value < 0.001) (Table 3).

### 3.3. In-Hospital Complications

Table 2 contrasts in-hospital outcomes for the dementia cohort against the non-dementia group. The dementia cohort presented a heightened risk of AKI (41% vs. 36%; aOR: 1.2, 95% CI 1.2–1.3, *p* < 0.001). Post-PSM findings mirrored this trend (Table 3). However, dementia patients showcased diminished risks in the following complications: acute liver failure (0.6% vs. 1.2%; aOR: 0.7, 95% CI 0.6–0.8), sudden cardiac arrest (2.6% vs. 3.3%; aOR: 0.9, 95% CI 0.8–0.9), vasopressor use (2.1% vs. 3.2%; aOR: 0.8, 95% CI 0.8–0.9), VTE (4.1% vs. 5.0%; aOR: 0.9, 95% CI 0.8–0.9), HD requirement (2.9% vs. 5.7%; aOR: 0.7, 95% CI 0.6–0.7), and mechanical ventilation (8.1% vs. 14.8%; aOR: 0.7, 95% CI 0.7–0.7), all with *p* < 0.001. Table 3 (post-PSM) reinforces these observations. Both cohorts exhibited comparable risks for cerebrovascular accidents and acute myocardial infarctions, around 2.6%. The aOR values were approximately 1.0 with no statistically significant differences, corroborated further by post-PSM analysis. In-hospital outcomes showed that dementia patients with COVID-19 had an average LOS of 8.5 days compared with 8.4 days for those without dementia, and this trend remained consistent post-PSM (difference of 0.5 days, *p* < 0.001). Moreover, the average hospitalization cost for dementia patients was USD 74,897, whereas it was USD 94,275 for the non-dementia group, with a noticeable cost difference of USD 6292 post-PSM (*p* < 0.001). Concerning discharge disposition, dementia patients were more frequently transferred to alternative healthcare facilities rather than given routine discharge (Table 2 and Table 3). 

### 3.4. Predictors of Mortality 

Using multivariate survival analysis, we identified several significant predictors for mortality in the cohort of COVID-19 patients with dementia. These included male gender (HR 1.1, 95% CI 1.1–1.2, *p* < 0.001), use of vasopressors (HR 1.1, 95% CI 1.03–1.3, *p* = 0.01), age 75–79 (HR 1.3, 95% CI 1.3–1.5, *p* < 0.001), age over 80 (HR 1.8, 95% CI 1.6–2.0, *p* < 0.001), as well as complications like AMI, AKI, use of both invasive and non-invasive mechanical ventilation, acute liver failure, and sudden cardiac arrest (Supplemental Figure S1).

## 4. Discussion

This study provides crucial evidence on the association between dementia and mortality in hospitalized COVID-19 patients. The significantly higher in-hospital mortality and AKI highlights the need for an individualized approach in this cohort. Further, dementia patients tend to have a longer hospital stay, lower hospital costs but higher need for post-discharge assistance (in terms of disposition).

Around 22% of hospitalized COVID-19 patients aged ≥65 years were identified with dementia. Existing literature highlights a clear association between dementia and increased severity of COVID-19 outcomes [12,13]. This elevated risk might be influenced by the challenges dementia patients face in adhering to social isolation, often due to their specific living conditions [2]. Although there is ample evidence of this link, the direct implications of dementia on COVID-19, and vice versa, remain under-investigated. Ghaffari et al. established an association between increased severity of COVID-19 and neurological complications, with dementia patients being particularly susceptible due to their pre-existing neurological deficits [13]. Such neurological challenges can cause uncharacteristic symptoms of COVID-19, leading to delayed diagnosis, and, consequently, increased in-hospital mortality. Further, delayed diagnosis and treatment may result from factors such as residence in assisted living facilities, reduced family visits due to pandemic-induced isolation, and evolving care objectives as their health deteriorates [3]. In essence, the heightened risk of severe COVID-19 outcomes in dementia patients is shaped by a combination of factors, including late diagnosis, social isolation, and a shift in patient priorities [13].

Previous data on dementia and COVID-19 is consistent with our findings regarding increased in-hospital mortality [14,15]. The pathophysiology of dementia heightens COVID-19 mortality through impaired cognition, compromised respiratory function, and challenges in symptom recognition [14]. Additionally, dementia-related immune dysregulation and chronic inflammation weaken viral defense, increasing susceptibility to severe respiratory complications and reducing resistance to COVID-19-induced physiological stress [16]. 

The coronavirus’s direct neuroinvasive capacity is uncertain, but potential mechanisms for inflammation involve cytokine interplay like interleukin-1 (IL-1) and interleukin-6 (IL-6), potentially collaborating with amyloid-induced type I interferon (IFN) in dementia [16]. This concealed impact of COVID-19 might exacerbate symptoms, intensifying COVID-19 severity in early-stage dementia. Moreover, pandemic stress could also hasten cognitive decline [16,17].

Our data indicate that COVID-19 patients with dementia had reduced occurrences of in-hospital vasopressor use, VTE, HD, and invasive mechanical ventilation. One plausible explanation could be patient and family preference for less aggressive interventions in this cohort, with an emphasis on comfort measures, particularly for patients with increased severity of disease. This is possibly due to the reduced physiological reserve and frailty commonly seen in dementia patients, leading to a more severe response to infections like COVID-19 despite the absence of common complications [18]. Several other studies have also demonstrated lower in-hospital complications in COVID-19 patients with dementia than in their non-dementia counterparts [14,15]. An Italian retrospective study aligns with our observations, noting that while dementia patients had an extended hospital stay and an elevated risk of kidney issues, infection, and in-hospital mortality, they faced lower complications from liver or heart disease and diabetes [14]. Despite this study not focusing exclusively on COVID-19 patients, the parallel finding of increased mortality juxtaposed with fewer comorbid complications reinforces our conclusion: dementia is an independent risk factor for severe outcomes, albeit with fewer secondary complications [15,19]. Additionally, cognitive impairment in dementia might contribute to delays in recognizing and treating symptoms of COVID-19, further exacerbating outcomes [18]. It is possible that the distinctive pathophysiology of COVID-19 in dementia patients underlies this observation. The altered immune response in dementia patients might counteract systemic inflammation from COVID-19 [14,15]. In the setting of COVID-19, the SARS-CoV-2 virus exploits ACE2 for cellular entry. ACE inhibitors and AT1R antagonists, often prescribed for conditions like hypertension, could potentially mitigate severe COVID-19 infection in dementia patients by preventing the cytokine storm and reducing the risk of end organ damage [20].

Patients with dementia are predominantly older and often present with concomitant health conditions such as hypertension, diabetes, and cardiovascular disease, which are major risk factors for AKI. Cognitive decline in dementia patients may hamper recognition of thirst, predisposing them to dehydration, and AKI in pre-hospital period [19,21]. The systemic inflammatory cascade, compounded by the direct nephrotoxicity attributed to the virus, can negatively affect renal function, thereby escalating AKI susceptibility [21,22]. Socio-environmental factors and quality of healthcare in nursing homes are important determinants of disease course as delayed diagnosis and management of COVID-19 can exacerbate the clinical picture, elevating the risk for mortality or AKI.

Our study revealed striking disparities in age and gender among COVID-19 patients with and without dementia. Women and individuals aged 80 years and above were disproportionately more likely to have dementia. These findings are consistent with the well-established risk factors for dementia, which include advanced age and female gender [23]. Hormonal changes in women and longer life expectancy are some of the reasons why women are more likely to develop dementia [23]. Similarly, African Americans and Latinos were overrepresented in the dementia cohort. This is in line with the broader public health literature, which has long highlighted the elevated risks of chronic illnesses among minority groups. These risks are often due to a complex interplay of factors, such as systemic inequalities, socio-economic conditions, and limited healthcare access [24]. With our robust sample size and accounting for confounders, our study offers a deeper and broader understanding of dementia patients with COVID-19 than previous research. We have spotlighted a multitude of significant mortality predictors among this group. Specifically, male gender, age brackets of 75–79 and 80 and above, the use of vasopressors, and in-hospital complications like AMI, AKI, both invasive and non-mechanical ventilation, acute liver failure, and sudden cardiac arrest emerged as critical markers. Such findings not only align with, but also augment, the existing literature [14,15]. Consistent with other studies, we identified male patients as particularly at risk, a reflection of findings suggesting greater vulnerability of men to severe COVID-19 outcomes. This vulnerability is often attributed to various factors, including pre-existing health conditions and hormonal differences [25,26]. Age stands out as another potent predictor, given the natural decline in physiological resilience and immune function with increasing age, further exacerbated by challenges innate to dementia [16,26]. The medical complications identified are all independently tied to increased mortality risks. These risks are amplified in dementia patients due to cognitive impairment and frail physiology and can also be indicative of COVID-19-related systemic inflammation and multi-organ involvement, worsening the prognosis [21].

In this study, we assessed the outcomes of hospitalized dementia patients with COVID-19. While our primary focus was on the direct effects of the virus on this group, it is imperative to acknowledge the precautionary measures in place during the study period. Care facilities for dementia patients adopted stringent sanitation practices and enforced visitor limitations. The adoption of telemedicine emerged as a preferred approach by which to minimize hospital visits, and potentially fewer patients required hospitalization as a result. Additionally, initial data indicate that vaccinations play a pivotal role in reducing the severity of disease manifestations, further limiting the necessity for hospital admissions [27].

Our study has some limitations, this is a retrospective study, relying on previously collected data, with its inherent potential for biases. The accuracy and completeness of ICD-10 codes from the NIS database could influence our results. Despite robust multivariate analyses, the possibility of residual confounding, especially from comorbid conditions that may influence outcomes, or frailty cannot be ruled out. While we controlled for a range of variables, the potential for residual confounding remains a pertinent issue, particularly in an observational study of this nature. Factors not captured in the dataset, such as medications, lifestyle choices, dementia subtypes or unrecorded medical conditions, might have impacted the outcomes. The lack of detailed clinical parameters, such as laboratory investigations and imaging, limits our ability to investigate the influence of disease severity or specific aspects of disease progression. Additionally, the absence of granular clinical data, such as specific patient histories and treatment regimens, limits the depth of our analysis. This lack of detailed information is a significant gap, as it could provide a more comprehensive understanding of the interaction between COVID-19 and dementia.

Another key limitation is the paradoxical finding of reduced complications in our dementia cohort. This intriguing outcome suggests the need for further research to explore underlying reasons, which may include variations in clinical management, patient health status, or other unmeasured factors. Finally, this study is focused on hospitalized COVID-19 patients and thus our findings may not be generalizable to patients managed in outpatient or home settings. Moreover, the exclusion of data on different dementia subtypes in our study restricts the scope of our analysis. As various forms of dementia have distinct pathologies and clinical trajectories, our findings might not be applicable across all dementia subtypes. Future research should differentiate between these to shed light on how each type specifically interacts with COVID-19.

Our data offer a foundation for future meta-analyses and trend comparisons from early to late pandemic phases, and can inform clinical trial enrollments and policymaking for dementia patients amidst pandemics. It also underscores the need for further research into COVID-19 and dementia to deepen our understanding of their relationship. The biological mechanisms behind the observed higher mortality and AKI in the dementia cohort are critical. Future studies should prioritize the inclusion of more detailed clinical data. This should encompass comprehensive clinical parameters, such as detailed patient medical histories, specific laboratory investigations, and imaging studies. Such granularity in clinical information would significantly enhance the validity and depth of research, enabling a more nuanced understanding of the interactions between COVID-19 and dementia, especially in determining the severity and progression of the disease.

Future research should aim to corroborate our findings through prospective cohort studies or randomized controlled trials. Additionally, the impact of co-morbid conditions, vaccination status, and various therapeutic interventions should be explored in more detail in order to provide a comprehensive risk profile for this vulnerable population. In particular, analyzing how detailed clinical profiles interact with the outcomes in COVID-19 patients with dementia would be invaluable. This includes exploring how specific treatments and interventions affect the progression of COVID-19 in patients with different stages and types of dementia, and how these factors may contribute to the variance in morbidity and mortality rates. Investigating how healthcare access and quality affect outcomes in dementia patients with COVID-19 would also be a valuable addition to the existing body of knowledge. Furthermore, understanding the impact of neuroinflammation in COVID-19 on the progression of disease and the effect of dementia on this pathway is essential. Investigations into various dementia subtypes’ differential impacts on COVID-19 outcomes can pave the way for preventive and therapeutic interventions tailored to each subtype. Through these expanded research avenues and by incorporating comprehensive clinical details, we can better tailor our understanding and management approaches to meet the specific needs of dementia patients during pandemics like that of COVID-19.

## 5. Conclusions

Our study provides compelling evidence for the significant impact of dementia on the mortality and clinical outcomes in hospitalized COVID-19 patients. We identified male sex, vasopressor use, and advanced age as substantial predictors of mortality, confirming their role as high-risk factors in this cohort. The study further unveiled racial and gender disparities, underlining the complex interplay of socio-demographic factors and clinical profiles in shaping health outcomes for dementia patients with COVID-19. While our data is aligned with existing literature, it also reveals unique insights, such as fewer in-hospital complications despite higher mortality, suggesting that dementia is an independent risk factor for severe COVID-19 outcomes. Clinicians should consider an individualized approach and targeted interventions for these patients. Ultimately, our study serves as a critical foundation for shaping both immediate clinical responses and long-term healthcare strategies, aiming to mitigate the severe impact of COVID-19 on one of the most vulnerable patient groups—those with dementia.

## Figures and Tables

**Table 1:** Baseline demographics.

Characteristics	COVID and Dementia−		COVID and Dementia+		*p* Value
	*n*	%	*n*	%	
*n* = 816,960	636,115	77.86	180,845	22.14	--
Gender (%)	*n*	%	*n*	%	<0.001
Female	298,580	46.94	100,555	55.6	
Male	337,535	53.06	80,290	44.4	
Mean Age Years (SD)	Mean	SD	Mean	SD	
Female	7616	7.54	8284	6.73	
AGE Groups (%)	*n*	%	*n*	%	<0.001
65–69	158,270	24.88	10,645	5.89	
70–74	155,240	24.4	19,820	10.96	
75–79	128,120	20.14	31,240	17.27	
≥80	194,485	30.57	119,140	65.88	
RACE (%)	*n*	%	*n*	%	<0.001
Asian or Pacific Islander	19,775	3.11	5295	2.93	
Black	99,140	15.59	30,195	16.7	
Hispanic	93,935	14.77	20,920	11.57	
Native American	4530	0.71	665	0.37	
Other	21,905	3.44	5690	3.15	
White	396,830	62.38	118,080	65.29	
MEDIAN HOUSEHOLD INCOME (%)	*n*	%	*n*	%	<0.001
≤49,999	208,790	32.82	55,130	30.48	
50K–64,999	179,450	28.21	46,710	25.83	
65K–85,999	142,335	22.38	40,745	22.53	
≥86k	105,540	16.59	38,260	21.16	
INSURANCE STATUS (%)	*n*	%	*n*	%	<0.001
Medicaid	18,710	2.94	3970	2.2	
Medicare	529,345	83.22	161,940	89.55	
No charge	690	0.11	150	0.08	
Other	17,215	2.71	4030	2.23	
Private insurance	64,095	10.08	9860	5.45	
Self-pay	6060	0.95	895	0.49	
HOSPITAL DIVISION (%)	*n*	%	*n*	%	<0.001
East North Central	110,515	17.37	30,240	16.72	
East South Central	46,700	7.34	12,530	6.93	
Middle Atlantic	98,055	15.41	28,225	15.61	
Mountain	40,065	6.3	7420	4.1	
New England	24,960	3.92	9575	5.29	
Pacific	60,885	9.57	19,790	10.94	
South Atlantic	124,210	19.53	40,210	22.23	
West North Central	45,810	7.2	10,450	5.78	
West South Central	84,915	13.35	22,405	12.39	
HOSPITAL BEDSIZE (%)	*n*	%	*n*	%	<0.001
Large	288,785	45.4	78,770	43.56	
Medium	184,900	29.07	56,265	31.11	
Small	162,430	25.53	45,810	25.33	
HOSPITAL TEACHING STATUS (%)	*n*	%	*n*	%	<0.001
Rural	76,190	11.98	17,730	9.8	
Urban nonteaching	123,675	19.44	36,115	19.97	
Urban teaching	436,250	68.58	127,000	70.23	
COMORBIDITIES (%)	*n*	%	*n*	%	
Coronary artery disease	172,895	27.18	48,970	27.08	0.392
Myocardial infarction	39,655	6.23	9425	5.21	<0.001
Essential hypertension	502,555	79	144,645	79.98	<0.001
Diabetes	286,660	45.06	69,910	38.66	<0.001
Cancer	39,265	6.17	6805	3.76	<0.001
Obesity	133,510	20.99	15,165	8.39	<0.001
Drug use	5105	0.8	795	0.44	<0.001
Smoking	195,245	30.69	41,325	22.85	<0.001
Alcohol	8695	1.37	1815	1	<0.001
Chronic pulmonary disease	169,840	26.7	40,125	22.19	<0.001
Peripheral vascular disease	38,480	6.05	12,125	6.7	<0.001
Chronic kidney disease	118,010	18.55	37,260	20.6	<0.001
Hypothyroidism	108,635	17.08	36,955	20.43	<0.001
Autoimmune	24,180	3.8	4805	2.66	<0.001
Depression	68,370	10.75	32,900	18.19	<0.001
AIDS	1510	0.24	295	0.16	<0.001

**Table 2:** Complications of patients with COVID-19 with and without dementia.

Characteristics	COVID and Dementia−		COVID and Dementia+		*p* Value
	*n*	%	*n*	%	
*n* = 816,960	636,115	77.86	180,845	22.14	--
Disposition (%)	*n*	%	*n*	%	<0.001
Against medical advice	4155	0.65	430	0.24	
Discharged alive unknown destination	525	0.08	330	0.18	
Home health care	116,605	18.33	25,010	13.83	
Routine	239,630	37.67	17,845	9.87	
Transfer other	136,925	21.53	91,450	50.57	
Transfer to short-term hospital	19,965	3.14	3945	2.18	
COMORBIDITIES (%)	*n*	%	*n*	%	
Acute liver failure	7480	1.18	1150	0.64	<0.001
	Adjusted odds ratio * = 0.66 (95% CI 0.57–0.77)				
Sudden cardiac arrest	20,805	3.27	4770	2.64	<0.001
	Adjusted odds ratio * = 0.87 (95% CI 0.81–0.94)				
Mean total hospitalization charge (USD)	USD 94,274.62		USD 74,896.89		<0.001
	Adjusted total charge * = USD 6075.18 lower for dementia+				
Mean length of stay (days)	8.43		8.50		<0.001
	Adjusted length of stay * = 0.64 days higher for dementia+				
In-hospital mortality (*n* = 160,145)	118,310	18.6	41,835	23.13	<0.001
	Adjusted odds ratio * = 1.17 (95% CI 1.13–1.2)				
Vasopressor use	20,260	3.18	3725	2.06	<0.001
	Adjusted odds ratio * = 0.81 (95% CI 0.75–0.88)				
AKI	226,915	35.67	74,950	41.44	<0.001
	Adjusted odds ratio * = 1.22 (95% CI 1.19–1.26)				
VTE	31,725	4.99	7395	4.09	0,001
	Adjusted odds ratio * = 0.90 (95% CI 0.84–0.95)				
Hemodialysis	35,945	5.65	5290	2.93	<0.001
	Adjusted odds ratio * = 0.67 (95% CI 0.62–0.72)				
Invasive mechanical ventilation	94,190	14.81	14,565	8.05	<0.001
	Adjusted odds ratio * = 0.68 (95% CI 0.66–0.72)				
Non-invasive mechanical ventilation	45,535	7.16	9315	5.15	<0.001
	Adjusted odds ratio * = 0.80 (95% CI 0.75–0.84)				
CVA	12,850	2.02	3660	2.02	0.598
	Adjusted odds ratio * = 1.02 (95% CI 0.94–1.12)				
Tracheostomy	6680	1.05	480	0.27	<0.001
	Adjusted odds ratio * = 0.43 (95% CI 0.35–0.54)				
AMI	16,625	2.61	4755	2.63	0.105
	Adjusted odds ratio * = 0.94 (95% CI 0.87–1.01)				

* Adjusted for age, hospital bed size, race, gender, hospital location, hospital teaching status, hospital region, median household income, expected primary payer (insurance status), and Elixhauser comorbidities.

**Table 3:** Complications of patients with COVID-19 with and without dementia after propensity score matching.

Characteristics	COVID and Dementia−		COVID and Dementia+		*p* Value
	*n*	%	*n*	%	
*n* = 361,690	180,845	50.00	180,845	50.00	--
Disposition (%)	*n*	%	*n*	%	<0.001
Against medical advice	835	0.46	430	0.24	
Discharged alive unknown destination	240	0.13	330	0.18	
Home health care	37,070	20.5	25,010	13.83	
Routine	49,300	27.26	17,845	9.87	
Transfer other	50,480	27.91	91,450	50.57	
Transfer to short-term hospital	4745	2.62	3945	2.18	
COMORBIDITIES (%)	*n*	%	*n*	%	
Acute liver failure	1680	0.93	1150	0.64	<0.001
	Adjusted odds ratio * = 0.68 (95% CI 0.58–0.81)				
Sudden cardiac arrest	5235	2.89	4770	2.64	0.006
	Adjusted odds ratio * = 0.88 (95% CI 0.8–0.96)				
Mean total hospitalization charge (USD)	USD 78,437.41		USD 74,896.89		<0.001
	Adjusted total charge * = USD 6291.82 lower for dementia+				
Mean length of stay (days)	7.93		8.50		<0.001
	Adjusted length of stay * = 0.49 days higher for dementia+				
In-hospital mortality (*n* = 80,010)	38,175	21.11	41,835	23.13	<0.001
	Adjusted odds ratio * = 1.16 (95% CI 1.12–1.2)				
Vasopressor use	4930	2.73	3725	2.06	<0.001
	Adjusted odds ratio * = 0.80 (95% CI 0.73–0.89)				
AKI	67,750	37.46	74,950	41.44	<0.001
	Adjusted odds ratio * = 1.21 (95% CI 1.17–1.25)				
VTE	8270	4.57	7395	4.09	0.002
	Adjusted odds ratio * = 0.89 (95% CI 0.83–0.96)				
Hemodialysis	7975	4.41	5290	2.93	<0.001
	Adjusted odds ratio * = 0.66 (95% CI 0.6–0.71)				
Invasive mechanical ventilation	20,690	11.44	14,565	8.05	<0.001
	Adjusted odds ratio * = 0.68 (95% CI 0.64–0.71)				
Non-invasive mechanical ventilation	11,450	6.33	9315	5.15	<0.001
	Adjusted odds ratio * = 0.77 (95% CI 0.73–0.83)				
CVA	3815	2.11	3660	2.02	0.299
	Adjusted odds ratio * = 0.95 (95% CI 0.85–1.05)				
Tracheostomy	1240	0.69	480	0.27	<0.001
	Adjusted odds ratio * = 0.41 (95% CI 0.32–0.52)				
AMI	4890	2.7	4755	2.63	0.320
	Adjusted odds ratio * = 0.95 (95% CI 0.87–1.05)				

* Adjusted for age, hospital bed size, race, gender, hospital location, hospital teaching status, hospital region, median household income, expected primary payer (insurance status), and Elixhauser comorbidities.

## Data Availability

We sourced our data from the 2020 edition of the national inpatient sample, part of the Healthcare Cost and Utilization project. As the NIS is accessible to the public, interested researchers can retrieve the data directly via the HCUP website (https://www.hcup-us.ahrq.gov/ accessed on 24 July 2023).

## Supplementary Materials

The following supporting information can be downloaded at: https://www.mdpi.com/article/10.3390/geriatrics9010007/s1, Figure S1: Mortality predictors in dementia and COVID-19+ patients; Table S1: ICD10 clinical modification codes; Table S2: Propensity matched: baseline patients characteristics 1:1 propensity matched variables.
