# Supplementary material for: Outcomes of COVID-19-Associated Hospitalizations in Geriatric Patients with Dementia in the United States: A Propensity Score Matched Analysis
Source: Geriatrics (Basel). 2024 Jan 5;9(1):7. doi: 10.3390/geriatrics9010007 (PMC10801614; doi:10.3390/geriatrics9010007)
Supplement: Supplementary file 1 [file geriatrics-09-00007-s001.zip › geriatrics-2641478-supplementary.pdf]

Supplemental Table S1. ICD 10 codes

Disease/Procedure	ICD-10 Codes
Covid 19	U071
Dementia	F0150,F0151,F0280,F0281,F0390,F0391, G3183,G300,G301,G308,G309,G3101,G3109
Drug induced dementia (excluded)	F1027,F1097,F1327,F1397,F1817,F1827,F1897,F1917,F1927,F1997
Vasopressor use	3E030XZ,3E033XZ,3E040XZ,3E043XZ,3E050XZ,3E053XZ,3E060XZ,3E063XZ
Mechanical ventilation invasive	0BH17EZ,0BH18EZ,5A1935Z,5A0945Z,5A0955Z
Non-invasive mechanical ventilation	5A09457,5A09458,5A09358,5A09557,5A09558,5A09357
Sudden cardiac arrest	I462,I468,I469
Hemodialysis	5A1D70Z,5A1D90Z,5A1D80Z,5A1D00Z,5A1D60Z
CKD	N181,N182,N1830,N1831,N1832,N184,N185,N189
AKI	N170,N171,N172,N178,N179,N990
Acute liver failure	K7200,K7201,K712
VTE	I82210,I82220,I82290,I82401,I82402,I82403,I82409,I82411, I82412,I82413,I82419,I82421,I82422,I82423,I82429,I82431,I82432, I82433,I82439,I82441,I82442,I82443,I82449,I82451,I82452, I82453,I82459,I82461,I82462,I82463,I82469,I82491,I82492,I82493, I82499,I824Y1,I824Y2,I824Y3,I824Y9,I824Z1,I824Z2,I824Z3,I824Z9, I82601,I82602,I82603,I82609,I82611,I82612,I82613,I82619,I82621, I82622,I82623,I82629,I82890,I8290,I82A11,I82A12,I82A13,I82A19, I82B11,I82B12,I82B13,I82B19,I82C11,I82C12,I82C13,I82C19,I1260, I2601,I2602,I2690,I2692,I2693,I2694,I2699,I2609
Smoking	F17,F172,F1720,F17200,F17201,F17203,F17208, F17209,F1721,F17210,F17211,F17213,F17218, F17219,F1722,F17220,F17221,F17223,F17228, F17229,F1729,F17290,F17291,F17293,F17298,F17299,Z87891
Tracheostomy	0B110F4,0B110Z4,0B113F4,0B113Z4,0B114F4,0B114Z4
AMI	I2101,I2102,I2109,I2111,I2119,I2121, I2129,I213,I220,I221,I228,I229,I214,I222
CAD	I2510,I25111,I25118,I25119,I252,I253,I254, I2541,I2542,I255,I256,I257,I2570,I25700, I25701,I25708,I25709,I2571,I25710,I25711,I25718, I25719,I2572,I25720,I25721,I25728,I25729,I2573,I25730, I25731,I25738,I25739,I2575,I25750,I25751,I25758,I25759,

	I2576,I25760,I25761,I25768,I25769
Chronic Pulmonary Disease	Elixhauser comorbidities index
Diabetes (2 types)	Elixhauser comorbidities index
AIDS	Elixhauser comorbidities index
Hypothyroidism	Elixhauser comorbidities index
Autoimmune	Elixhauser comorbidities index
Dementia	Elixhauser comorbidities index
Depression	Elixhauser comorbidities index
Lymphoma	Elixhauser comorbidities index
Leukemia	Elixhauser comorbidities index
Metastatic Cancer	Elixhauser comorbidities index
Solid Tumor Without Metastasis (2 types)	Elixhauser comorbidities index
Obesity	Elixhauser comorbidities index
Drug Abuse	Elixhauser comorbidities index
Hypertension (2 types)	Elixhauser comorbidities index
PAD Peripheral vascular disease	Elixhauser comorbidities index
Alcohol	Elixhauser comorbidities index
Cerebrovascular accident	I6000,I6001,I6002,I6010,I6011,I6012,I602,I6020,I6021,I6022,I6030,I6031,I6032, I604,I6050, I6051,I6052,I606,I607,I608,I609,I610,I611,I612,I613,I614,I615,I616,I618, I619,I6200, I6201,I6202,I6203,I621,I629,I6300,I63011,I63012,I63013,I63019, I6302,I63031,I63032,I63033,I63039,I6309,I6310,I63111,I63112,I63113,I63119, I6312,I63131,I63132, I63133,I63139,I6319,I6320, I63211,I63212,I63213,I63219,I6322,I63231,I63232,I63233,I63239,I6329,I6330,I63311, I63312,I63313, I63319,I63321,I63322,I63323,I63329,I63331, I63332,I63333,I63339,I63341,I63342,I63343,I63349,I6339,I6340,I63411,I63412,I63413, I63419,I63421,I63422, I63423,I63429,I63431,I63432,I63433,I63439,I63441,I63442,I63443,I63449,I6349, I6350,I63511,I63512,I63513,I63519,I63521,I63522,I63523,I63529,I63531,I63532,I63533, I63539,I63541,I63542,I63543,I63549,I6359,I636,I638,I6381,I6389,I639

Table S2. Baseline demographics after propensity score matching.

Characteristics	Covid & Dementia-		Covid & Dementia+		P value
	N	%	N	%	
N = 361,690	180845	50,00	180845	50,00	--
**Gender (%)**	N	%	N	%	<0.001
Female	97415	53,87	100555	55,6	
Male	83430	46,13	80290	44,4	
**Mean Age Years (SD)**	**Mean**	**SD**	**Mean**	**SD**	
Female	82,48	6,76	82,84	6,73	
Male	81,44	7,09	81,08	7,08	
**AGE Groups (%)**	N	%	N	%	0,002
65-69	10635	5,88	10645	5,89	
70-74	20530	11,35	19820	10,96	
75-79	30945	17,11	31240	17,27	
>=80	118735	65,66	119140	65,88	
**RACE (%)**	N	%	N	%	<0.001
Asian or Pacific Islander	4800	2,65	5295	2,93	
Black	28870	15,96	30195	16,7	
Hispanic	19540	10,8	20920	11,57	
Native American	590	0,33	665	0,37	
Other	5580	3,09	5690	3,15	
White	121465	67,17	118080	65,29	
**MEDIAN HOUSEHOLD INCOME (%)**	N	%	N	%	<0.001
<=49,999	55975	30,95	55130	30,48	
50K-64,999	47740	26,4	46710	25,83	
65K-85,999	40645	22,48	40745	22,53	
>=86k	36485	20,17	38260	21,16	

**INSURANCE STATUS (%)**	**N**	**%**	**N**	**%**	<0.001
Medicaid	3635	2,01	3970	2,2	
Medicare	163590	90,46	161940	89,55	
No charge	130	0,07	150	0,08	
Other	3965	2,19	4030	2,23	
Private Insurance	8875	4,91	9860	5,45	
Self-pay	650	0,36	895	0,49	
**HOSPITAL DIVISION (%)**	**N**	**%**	**N**	**%**	<0.001
East North Central	39795	22,01	30240	16,72	
East South Central	8195	4,53	12530	6,93	
Middle Atlantic	45520	25,17	28225	15,61	
Mountain	6290	3,48	7420	4,1	
New England	14055	7,77	9575	5,29	
Pacific	10645	5,89	19790	10,94	
South Atlantic	28495	15,76	40210	22,23	
West North Central	13410	7,42	10450	5,78	
West South Central	14440	7,98	22405	12,39	
**HOSPITAL BEDSIZE (%)**	**N**	**%**	**N**	**%**	<0.001
Large	78280	43,29	78770	43,56	
Medium	52550	29,06	56265	31,11	
Small	50015	27,66	45810	25,33	
**HOSPITAL TEACHING STATUS (%)**	**N**	**%**	**N**	**%**	<0.001
Rural	22085	12,21	17730	9,8	
Urban nonteaching	31580	17,46	36115	19,97	
Urban teaching	127180	70,33	127000	70,23	
**COMORBIDITIES (%)**	**N**	**%**	**N**	**%**	
CAD	53965	29,84	48970	27,08	0,392
MI	12160	6,72	9425	5,21	<0.001
HTN	145005	80,18	144645	79,98	0,133
Diabetes (2)	70860	39,18	69910	38,66	0,001

Cancer (5)	6380	3,53	6805	3,76	<0.001
Obesity	14695	8,13	15165	8,39	0,004
Drug Abuse	530	0,29	795	0,44	<0.001
Smoking	52425	28,99	41325	22,85	<0.001
Alcohol	1610	0,89	1815	1	<0.001
Chronic Pulmonary Disease	41105	22,73	40125	22,19	<0.001
Peripheral Vascular Disease	11510	6,36	12125	6,7	<0.001
CKD	41235	22,8	37260	20,6	<0.001
Hypothyroidism	36435	20,15	36955	20,43	<0.001
Autoimmune	4410	2,44	4805	2,66	<0.001
Depression	28175	15,58	32900	18,19	<0.001
AIDS	225	0,12	295	0,16	0,002

Supplemental Figure S1. Mortality Predictors in COVID-19 and Dementia + Patients

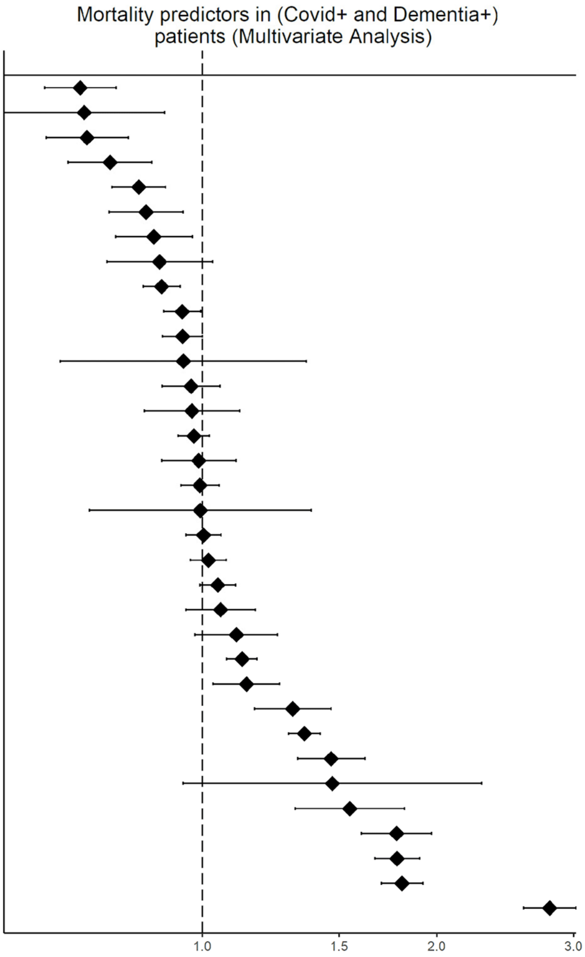

Variable	HR	CI	p-value
Venous thromboembolism	0.70	(0.63–0.77)	<0.001
CMR_ALCOHOL	0.71	(0.56–0.89)	0.004
RACE:Black	0.71	(0.63–0.80)	<0.001
RACE:Hispanic	0.76	(0.67–0.86)	<0.001
Obesity	0.83	(0.77–0.90)	<0.001
Hemodialysis	0.85	(0.76–0.94)	0.003
RACE:White	0.87	(0.77–0.97)	0.013
RACE:Other	0.88	(0.75–1.03)	0.113
Hypertension	0.89	(0.84–0.94)	<0.001
Smoke	0.94	(0.89–1.00)	0.034
Depression	0.94	(0.89–1.00)	0.051
RACE:Native American	0.95	(0.66–1.36)	0.761
Peripheral vascular disease	0.97	(0.89–1.05)	0.440
Autoimmune	0.97	(0.84–1.12)	0.667
Diabetes	0.97	(0.93–1.02)	0.280
Cancer	0.99	(0.89–1.10)	0.853
Hypothyroidism	0.99	(0.94–1.05)	0.814
Drug abuse	0.99	(0.72–1.38)	0.971
Chronic pulmonary disease	1.00	(0.95–1.06)	0.901
Chronic kidney disease	1.02	(0.96–1.07)	0.515
Coronary artery disease	1.05	(0.99–1.10)	0.087
Myocardial infarction	1.06	(0.95–1.17)	0.305
agegroup:70-74	1.11	(0.98–1.25)	0.105
Male	1.12	(1.08–1.18)	<0.001
Vasopressor use	1.14	(1.03–1.26)	0.010
agegroup:75-79	1.31	(1.17–1.46)	<0.001
Acute kidney injury	1.35	(1.29–1.42)	<0.001
Acute myocardial infarction	1.46	(1.32–1.62)	<0.001
AIDS	1.47	(0.94–2.29)	0.088
Acute liver failure	1.55	(1.32–1.82)	<0.001
agegroup:80andover	1.78	(1.60–1.97)	<0.001
Non-invasive mechanical ventilation	1.78	(1.67–1.90)	<0.001
Invasive mechanical ventilation	1.81	(1.70–1.92)	<0.001
Sudden cardiac arrest	2.80	(2.59–3.02)	<0.001
